# Study of 138 vulvar lichen sclerosus patients and the malignant risk transformation

**DOI:** 10.61622/rbgo/2024rbgo62

**Published:** 2024-07-26

**Authors:** Bruna Obeica Vasconcellos, Susana Cristina Aidé Viviani Fialho, Isabel Cristina Chulvis do Val Guimarães, Caroline Alves de Oliveira Martins, José Rodrigo de Moraes, Rita Maira Zanine, Julia Correa Cardoso Guimarães, Faustino Pérez-López

**Affiliations:** 1 Universidade Federal Fluminense Niterói RJ Brazil Universidade Federal Fluminense, Niterói, RJ, Brazil.; 2 Universidade Federal do Paraná Curitiba PR Brazil Universidade Federal do Paraná, Curitiba, PR, Brazil.; 3 University of Zaragoza Faculty of Medicine Zaragoza Spain University of Zaragoza Faculty of Medicine, Zaragoza, Spain.

**Keywords:** Carcinogenesis, Vulvar cancer, Vulvar lichen sclerosus, Risk assessment, Risk factors

## Abstract

**Objective:**

To report the prevalence of malignant transformation of vulvar lichen sclerosus (VLS) and possible risk factors.

**Methods:**

This is a cohort study with data analysis from medical records of 138 patients with histological diagnosis of VLS registered at the Vulvar Pathology Outpatient Clinic of the University Hospital, between 2007 and 2017. Predominance of risk factors was performed using logistic regression analysis. The variables studied were the length of follow-up, age, regular or irregular follow up; presence of symptoms (dyspareunia, pruritus and/or vulvar burning); histology characteristics, the presence of epithelial hyperplasia; and the presence of autoimmune diseases.

**Results:**

There were 138 patients included in the study, and among them five progressed to malignant transformation. The patients had a median age of 59 years and 83% were symptomatic. The most frequent symptom was itching with 72%. Autoimmune diseases were present in 11.6%, the most prevalent being thyroid disease. All five case of malignant transformation (0.6%) had an irregular follow up. The logistic regression analysis was used among the studied variables, and no statistical significance was found among them (p ≥ 0.05). The relationship between hyperplasia and the clinical outcome of malignant transformation, in which non-significant but acceptable p value close to 0.05 was observed.

**Conclusion:**

The prevalence of malignant transformation in patients with VLS was 0.6%, and common factors were the lack of adherence to medical treatments and the loss of follow-up.

## Introduction

Vulvar lichen sclerosus (VLS) is a chronic dermatological pathology of benign character and undetermined etiology, which may be associated with certain factors, such as autoimmune disorders, genetic alterations, states of hypoestrogenism, infections, and repeated traumas in the affected area.^(1-3)^This disease is reported predominantly in white women, in pre-pubertal and post-menopausal periods, with a rate that can vary from 6 to 10 women for each affected man.^(1)^It has a predominance in the anogenital region (83%-98%), but it may have extragenital involvement. The described symptoms are pruritus (most frequent), irritation, dyspareunia, and dysuria. On physical examination, there are hypochromic spots in the vulvar region, effacement of the labia minora and clitoral entrapment. This set of signals and symptoms may be responsible for high morbidity and reduced quality of life.^(4-6)^In addition, there is the possibility of malignant transformation (5%), which may initially evolve into a pre-malignant lesion, called differentiated vulvar intraepithelial neoplasia with progression to vulvar squamous cell carcinoma, with the histological type in this route of carcinogenesis being squamous cell carcinoma. invasive keratinizing.^(1,3,7,8)^

This study evaluate the prevalence of vulvar cancer among patients with VLS and the risk factors for the occurrence of malignant transformation in a Brazilian University Reference Center.

## Methods

This is cohort study of 138 women with a histopathological diagnosis of VLS. The convenience sample was performed according to the number of patients with a histopathological diagnosis of VLS being followed at the Vulvar Pathology Outpatient Clinic of a University Hospital 2007 to 2017. The patients voluntarily consented and agreed to participate in the study by signing the Free and Informed Consent Form.

Information was collected at the time of the medical appointment at the Vulvar Pathology Outpatient Clinic at University Hospital or through the medical records. The numerical variables studied were length of follow-up (evaluated by months, defined as the time when patient was diagnosed with VLS in the outpatient clinic to the final date of data collection) and age. Related to categorical variables: adequate follow-up (regular and irregular follow-up is considered to be a patient who, after responding to treatment, had her annual check-ups until data collection); the presence of symptoms (dyspareunia, pruritus, and/or vulvar burning); regarding histology, the presence of epithelial hyperplasia; and the presence of autoimmune diseases.

For the descriptive measurements of the ages of the 138 patients, the Shapiro-Wilk test was applied. A logistic regression test was performed to identify a possible relationship between the studied variables and the outcome of interest (vulvar cancer). The variables analyzed were age, follow-up time, outcome, response to treatment, symptoms, age, and presence of autoimmune diseases. Data were analyzed using the SPSS 23.0 statistical package. The result of p < 0.05 was considered significant. In addition, Fisher’s exact test was used to assess a possible association between categorical variables and outcomes.

The non-paparametric Mann-Whitney test or the Student’s t-test for independent samples was adopted to compare the values of age and follow-up time between two groups of patients, that is, between patients with regular versus irregular follow-up, and also among patients with presence versus absence of vulvar cancer. The normality of both age of the patients how much of their follow-up times was assessed through the Shapiro-Wilk. Additionally, univariate logistic models were adjusted (crude analysis) for the study outcome, in order to estimate the odds ratios of patient has vulvar cancer, with their 95% confidence intervals and p- Wald test values.

The study was approved by the Ethics Committee of the Faculty of Medical Sciences, with the number 5.083.505, under the recommendations of Resolution No. 466 of December 12, 2012, of the National Health Council, which establishes Guidelines and Regulatory Norms for Research Involving Human Beings and guides the evaluation of ethical issues of research projects.

## Results

### Clinical characteristics

**Figure 1:**
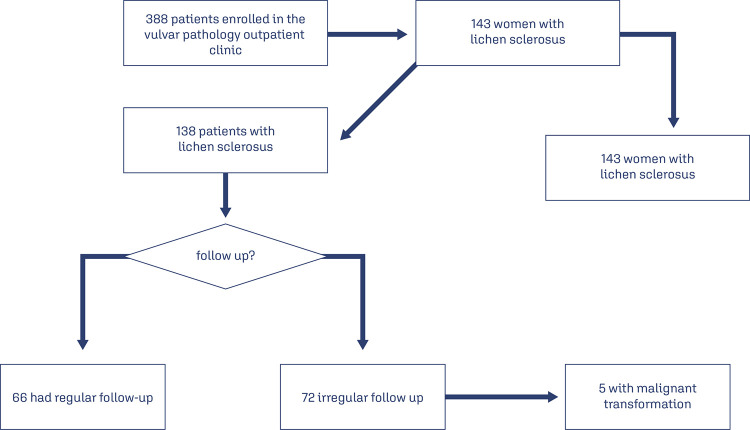
Participants in the study

Of the 388 women whose data was analyzed over the 10-year period that was evaluated 143 had VLS. Five participants were excluded due to lack of access to their medical records (Figure 1).

**Figure 2:**
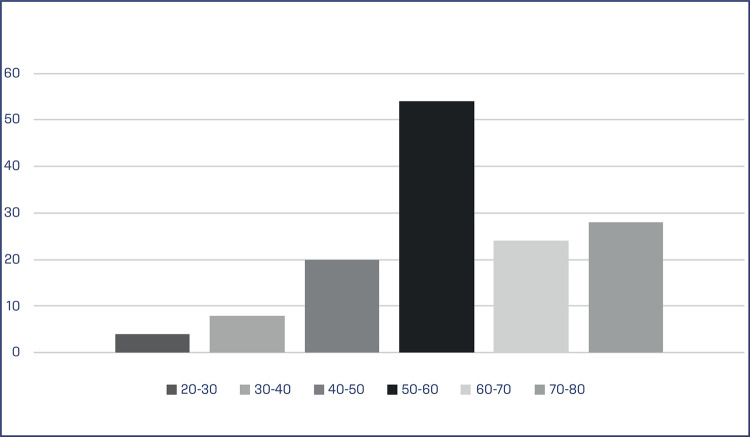
Patient with VLS according to age at diagnosis

According to the Shapiro-Wilk test, the age distribution of the patients has an approximately normal distribution (p-value=0.093). The patients have a mean age of 58.2 years and a standard deviation of 12.3 years. Additionally, patients have a median age of 59 years and an interquartile range of 50 to 68 years. (Table 1). The most common age group observed among patients with VLS was between 50-60 years (39%) of age, followed by the range of 70-80 years (20%) (Figure 2).

**Table 1:** Characteristics of women with VLS

Characteristics	Median	Group most common VLS(%)/ n(%)	Standard derivation
Age	59	50-60(39)	50 a 68 age (interquartile rage)
Characteristics			
Symptoms			
Asymtomatic		26(17)	
Symptomatic		112(83)	
Itching		83(72)	
Dyspareunia		12(15)	
Burning		13(13)	
Autoimmune diseases		16(11.6)	
Thyroid disease		4	
Psoriasis		2	
Type I diabetes mellitus		2	
Others		1 for each	
Follow up			
Adequate		66(48)	
Inadequate		72(52)	
Malignant transformation		5(0.6)	
Epithelial hyperplasia			
Yes		3(2)	
No		110(80)	
Unknown		25(18)	
Follow up time	Average (months)	Minimum/maximum (months)	
	54.2	1-143	

Regarding symptoms prior to the treatment, from 138 patients, it was observed that 26(17%) were asymptomatic, while 112(83%) were symptomatic. The most frequent symptom was itching with 83(72%), followed by dyspareunia with 12(15%) and burning with 13(13%) (Table 1).

Among the 138 patients with VLS, 16(11.6%) had autoimmune diseases. Among them, the most prevalent were thyroid disease (4), followed by psoriasis (2) and type 1 diabetes mellitus (2), with only one patient for each of the following pathologies: rheumatic fever, fibromyalgia, idiopathic thrombocytopenia purpura and bullous pemphigus (Table 1). The mean follow-up time was 54.2 months, ranging from 1 to 143 months. Among the follow-up of patients with VLS, it was observed that 66(48%) of the patients maintained adequate follow-up, while in 72(52%) the follow-up was inadequate. All 5 cases of malignant transformation (0.6%) were observed in patients with inadequate follow-up (Figure 1). The prevalence of malignant transformation found in patients with VLS was 0.6 % (Table 2). In addition, in ten years of patients’ follow-up had vulvar cancer during the period. All cases with the histological type of invasive keratinizing squamous cell carcinoma. The patients in question were followed up for 96, 81, 30, 57, and 38 months.

**Table 2:** Patient with malignant transformation

Patients	Age	Epithelial hyperplasia	Autoimmune diseases	Symptoms	Clinical conditions	Treatment	Follow up time	Follow up
A	72	Yes	Yes	icthing	color change	Yes	104	No
B	59	No	No	icthing	color change	Yes	86	No
C	59	No	No	icthing	color change, atrophy structures	Yes	81	No
D	66	There was no data for evaluation	Yes	No	color change	Yes	84	No
E	60	There was no data for evaluation	Yes	icthing	color change, atrophy structures, lichenification	Yes	79	No

Using the logistic regression test it was not possible to observe a statistically significant association between the studied variables (numerical and categorical) and the vulvar cancer outcome. In the group of patients who had malignant transformation, itching was the most prevalent symptom, and the most observed chances were color chase (4), atrophy (2) and lichenification (2). The autoimmune diseases observed in this group were scklen cell anemia (1), scleroderma (1) and hypothyroidism (1). Finally, all report improvement in symptoms after treatment, however all had failure to follow up (Table 3).

**Table 3:** Irregular follow up vs Regular follow up

Features	Irregular follow up n=72 n(%)	Regular follow – up n=66 n(%)	p-value
Hiperplasia			0.934^*^
Yes	2(2.8)	1(1.5)	
No	56(77.8)	53(80.3)	
Uninformed	14(19.4)	12(18.2)	
Age (years)			0.298^**^
Média	59.3(12.8)	61.5(11.1)	
Autoimmune Diseases			0.782^*^
Yes	8(11.1)	6(9.1)	
No	64(88.9)	60(90.9)	
Symptoms			0.839^*^
Asymptomatic	17(23.6)	14(21.2)	
Symptomatic	55(76.4)	52(78.8)	
Types of symptoms (Symptomatic)			
Itching	38(52.8)	43(65.2)	
Burning	0(0.0)	1(1.5)	
Dyspareunia	1(1.4)	0(0.0)	
Itching-Burning	6(8.3)	0(0.0)	
Itching-Dyspareunia	6(8.3)	6(9.1)	
Burning-Dyspareunia	2(2.8)	0(0.0)	
Burning-Dyspareunia-Itching	2(2.8)	1(1.5)	
Uninformed	0(0.0)	1(1.5)	
Clinical condition			0.491^*^
1 of 3 clinical signs	21(29.2)	25(37.9)	
2 of 3 clinical signs	20(27.8)	14(21.2)	
3 clinical signs	31(43.0)	27(40.9)	
Treatment			0.621^*^
Yes	69(95.8)	65(98.5)	
No	3(4.2)	1(1.5)	
Clinical Improvement			0.018^*^
Yes	63(87.5)	65(98.5)	
No	9(12.5)	1(1.5)	
Vulvar Cancer			0.059^*^
Yes	5(6.9)	0(0.0)	
No	67(93.1)	66(100.0)	
Follow up time (months)			0.378^***^
Media	54.2(28.3)	50.6(27.8)	

* p-value of Fishers test; ** p-value of t-Student test; *** p-value of Mann-Whitney test

With the exception of response to treatment/clinical improvement (p-value < 0.05), there was no significant difference between the distribution of characteristics of patients with regular follow-up and patients with an irregular follow up. The presence of epithelial hyperplasia was analyzed in the biopsy results and was found in 3 cases (2%) In 15 women (18%) the results were unknown due to lack of histological description and 110 (80%) were negative (Table 1). Among the positive cases, one patient had an outcome of malignant transformation (Table 3). Using both Fisher’s tests and logistic regression, between hyperplasia and the clinical outcome of malignant transformation, non-significant but acceptable p values close to 0.05 were observed.

## Discussion

The incidence of VLS is rare, varying around 1:300 and 1:1000 cases in women; however, its exact prevalence is still unknown.^(8-10)^ Studies show that VLS can be found at any stage of a woman’s life, however it tends to have two onset peaks: pre-pubescent and peri- or postmenopausal women, which characterizes a bimodal distribution.^(3,4,6)^ Powell et al.^12^ carried out a cohort study with 17,000 women followed for a long-term period and verified a positive relationship between age and the occurrence of VLS, with an incidence rate of 14 per 100,000 women/year, with ages ranging between 50 and 59 years.^(11-14)^ Our study found similar results in which the most prevalent age group was 50 to 60 years old, followed by the range between 70 and 80 years old.^(12)^

The most common symptom of VLS is itching (93%) and there may be pain as a result of excoriation or fissure. However, occasionally, the disease can be completely asymptomatic, discovered by chance by the patient or by the doctor during the gynecological examination.^(15,16)^ This situation can be dangerous, as asymptomatic disease may not be noticed until the carcinoma appears. In the present study, most patients were symptomatic (81.2%) and pruritus was the most prevalent symptom, corroborating the literature.

The exact etiopathogenesis of VLS is not known, but several theories have been documented. There are indications that one of its causes is immune, being one of the most accepted since linked to other autoimmune diseases, among them thyroid disease, pernicious anemia, alopecia areata, vitiligo, type 1 diabetes mellitus, primary biliary cirrhosis, and systemic lupus erythematosus. The two most frequently seen diseases were autoimmune thyroid disease and vitiligo.^(1,17,18)^ In the present study, 11.6% of patients had autoimmune diseases, the most prevalent being thyroid disease, followed by type 1 diabetes mellitus and systemic lupus erythematosus, corroborating data found in the literature.^(19,20)^

Analyzing four studies, it was observed that the malignancy potential for patients with VLS is around 2% to 6%.^(21)^ A recent study found that the only differentiating factor between those who developed malignant progression from those who did not was the consistent suppression of the disease with continuous topical corticosteroid therapy. This treatment may represent a very important strategy, since it is believed that its continuous use would stabilize the inflammation caused by VLS, and therefore reduce the chance of evolution with malignant transformation, with regular follow-up being essential.

Out of the 138 patients analyzed, it was noted that the five cases of malignant transformation were not in regular follow-up, that is, they did not keep their annual appointments and were not using high-potency topical corticosteroids (clobetasol proprionate) on a regular basis (weekly application) for disease stabilization, probably due to difficulties in scheduling, as well as social and economic factors, in addition to the lack of risk perception about lichen sclerosus disease. Despite this, the follow-up, whether adequate or not, did not show a significant risk for malignant transformation in the sample of the present study. Irregular follow-up contributes to the lack of diagnosis of the pre-malignant disease, causing patients to only seek medical care due to the symptoms caused by the progression to cancer. This demonstrates the need for further studies on the subject, even with limited sample and study time.

A retrospective study in a hospital in Oxfordshire, in 2004, of 976 women with VLS revealed a risk of incidence of neoplasms of 3.5%.^(13)^ A cohort including 3038 women from the provinces of Noord-Holland and Flevoland in the Netherlands diagnosed with VLS between 1991 and 2011 showed a cumulative incidence of squamous cancer of 6.7%.^(11)^ In the Leis et al.^(17)^ systematic review, the absolute risk of developing squamous cancer in patients with VLS, the authors found a risk of up to 21.88%. However, the vulvar cancer risk is quite variable depending on the population studied, diagnostic procedures, and duration of follow-up.^(21)^ In the present study, malignant transformation was 0.6%.

Studies suggest that the risk of malignancy is reduced in properly diagnosed and treated VLS.^(5,13)^A prospective cohort study with 507 adult women with VLS compared patients who adhered to treatment and those who did not. Diagnosis of biopsy-proven squamous cell carcinoma or vulvar intraepithelial neoplasia during follow-up was zero in treatment-adherent patients versus 7(4.7%) non-adherent patients (p < 0.001), demonstrating that corticosteroid treatment kept the skin closer to normal, resulting in minimal scarring and reducing the risk of cancer.^(18-20)^ In the present study, it was observed that patients with malignant transformation (0.6%) were not undergoing continuous treatment and/or adequate follow-up.

According to the review done by Singh et al. ^22^the cumulative probability of progression to neoplasia increases from 1.2% in 24 months to 36.8% in 300 months. In addition, the mean progression-free survival was significantly lower in elderly women (≥ 70 years) when compared to women from the fourth decade of life (p = 0.003). In that same article, it was shown that the cumulative probability of progression to neoplasia increases from approximately 1% at two years of follow-up to almost 37% at 25 years of follow-up. This therefore shows the importance of lifetime follow-up. In the present study, in ten years of patients’ follow-up, only five had cancer during the period. The patients in question were followed up for 96, 81, 30, 57 and 38 months.

Histologically in VLS, flattening with rectification of the dermal papillae with or without epithelial hyperplasia, inflammatory infiltrate and homogenization of the underlying collagen are observed. In a study at the Tarnier Hospital in Paris in 1990, in a series of 48 cases of VLS associated with invasive squamous cell carcinoma, Leibowitch et al.^(23)^showed that in 83% of the cases, carcinoma has been developed from epithelial hyperplasia and that in 70% of cases of hyperplasia, atypia was observed. These areas of hyperplasia with or without atypia may have a potential for malignant transformation in VLS.^(1,11,13,14)^ In the present study, among the positive cases for epithelial hyperplasia, one of them evolved with the outcome of malignant transformation. Still in logistic regression, considered possible contributing factors for malignancy, only epithelial hyperplasia showed non-significant but acceptable p values close to 0.05 were observed.

Because it is a low-prevalence disease, even in a long follow-up, we had a limited number of participants, not being able to reach a conclusion about the correlation between risk factors and clinical outcome.

## Conclusion

The prevalence of malignant transformation in patients with VLS was around 0.6%, and the common factor was the loss of follow-up. No statistically significant associations were observed between the studied variables and the occurrence of malignant transformation in women living in Rio de Janeiro area.
